# Green tea catechins inhibit the endonuclease activity of influenza A virus RNA polymerase

**DOI:** 10.1371/currents.RRN1052

**Published:** 2009-10-13

**Authors:** Takashi Kuzuhara, Yuma Iwai, Hironobu Takahashi, Dai Hatakeyama, Noriko Echigo

**Affiliations:** ^*^Laboratory of Biochemistry, Faculty of Pharmaceutical Sciences, Tokushima Bunri University; ^‡^Institute of Pharmacognosy Tokushima Bunri University and ^§^Tokushima Bunri University

## Abstract

The influenza A RNA polymerase possesses endonuclease activity to digest the host mRNA. Thus this endonuclease domain can be a target of anti-influenza A virus drug. Here we report that green tea catechins inhibit this viral endonuclease activity and that their galloyl group is important for their function. Docking simulations revealed that catechins with galloyl group fit well into the active pocket of the endonuclease domain to enable stable binding. Our results provide useful data that make it possible to refine and optimize catechin-based drug design more readily for stability.

## Introduction

     In 1918, a pandemic expansion of influenza A virus killed more than 10 million people worldwide [1] and the prevention of future expansions of this virus is therefore an important endeavor. The emergence of new swine-origin H1N1 influenza A this year emphasizes this further as it represents a serious global health issue [2]. For the prevention and control of such new influenza outbreaks, the development of new antiviral drugs is critical [2]. Although inhibitors of neuraminidase are widely used as anti-influenza A drugs, some adverse effects of these agents and also the emergence of drug-resistant viruses have now been reported [3] [4]. The influenza A virus genome comprises a negative RNA strand, the transcription and replication of which requires the activity of an RNA-dependent RNA polymerase [5]. This viral enzyme is highly conserved and thus represents a very promising target for anti-viral drug development. The influenza A virus RNA-dependent RNA polymerase is composed of three subunits, PA, PB1 and PB2 [6] [7] [8], and synthesizes viral mRNAs using short capped primers derived from host cellular pre-mRNAs. These molecules are cleaved after 10-13 nucleotides by an endonuclease in the N-terminal domain of the PA subunit. This unique endonuclease activity is thus essential for the influenza virus to propagate and should also be regarded as a potentially important target for anti-viral drug design. Yuan *et al.* and Dias *et al.* have recently shown that the N-terminal domain of the PA subunit contains the endonuclease active site in its tertiary structure and that this domain also has RNA and DNA endonuclease activity [6] [8]. This reported information regarding the tertiary structure of PA can be used to refine possible endonuclease inhibitor candidates.

     Green tea is a popular and healthy beverage in Japan. Most of the active components of green tea are assumed to be the catechins, because they have been shown to possess a variety of properties in in vitro cell cultures and also in vivo, including anti-oxidant, anti-cancer and DNA-binding activities [9] [10]. (-)-Epigallocatechin gallate (EGCG), (-)-epigallocatechin (EGC), (-)-epicatechin gallate (ECG), (-)-epicatechin (EC), (-)-gallocatechin gallate (GCG) and (+)-catechin are the major components of green tea polyphenols [9], and EGCG is one of the major components of green tea catechins (chemical structures are shown in Fig. 1) [9]. In addition, Song et al., have reported that EGCG at relatively high doses is an inhibitor of influenza A virus replication and also of viral RNA synthesis in cells [11]. They concluded from these findings that the antiviral effects of catechins on influenza are mediated via alterations in the physical properties of the viral membrane [11]. However, it has not yet been demonstrated how to refine and to optimize EGCG for use as an anti-influenza viral drug. The objective of this study is to investigate whether green tea catechins inhibit influenza A virus endonuclease and which chemical groups of catechin are important for this activity. In this study, we report that the several kinds of green tea catechins can inhibit the endonuclease activity of the PA subunit of the influenza A virus and be refined for a candidate of the anti-viral drug through *in silico* calculation.

## Materials & Methods

### Expression and purification of the N-terminal endonuclease domain protein of influenza A virus RNA dependent RNA polymerase

     The influenza (A/PR/8/34) H1N1 RNA polymerase PA plasmid, pBMSA-PA, was sourced from the DNA Bank, Riken BioResource Center (Tsukuba, Japan; originally deposited by Dr. Susumu Nakada). The cDNA fragment corresponding to the influenza A virus RNA polymerase PA N-terminal domain (residues 1 - 220) was amplified by PCR from pBMSA-PA (Riken) using the primers: PA endonuclease forward NdeI, GCCGTTCATATGGAAGATTTTGTGCGACAA; and PA endonuclease reverse BamHI, GCCGTTGGATCCTATTGGTCGGCAAGCTTGCG. The amplified product was then subcloned into the pET28a(+) plasmid (Novagen, Madison, WI) at the NdeI and BamHI restriction sites. The construct was then introduced into BL21-CodonPlus (Stratagene, La Jolla, CA) E. coli cells. The induction of 6x his-tagged recombinant protein expression from this construct was achieved by the addition of isopropyl beta-D-thiogalactopyranoside (IPTG) in TBGM9 medium and this was followed by purification using Ni^2+^-agarose. The recombinant protein was further purified to near homogeneity using a HiTrap™ Q FF column (GE Healthcare, Buckinghamshire, UK) with the Akta™ prime plus system (GE Healthcare).

### Endonuclease activity assay of the N-terminal domain protein of PA subunit of influenza A RNA dependent RNA polymerase

     Endonuclease assays of the influenza A RNA polymerase PA subunit were performed essentially as described by Dias et al. [6] [8] with some modifications. Briefly, we modified the pH conditions from 8.0 to 7.3 and used 1 μg of M13mp18 single stranded circular phage DNA as the assay substrate. We added 0.35 μg of recombinant N-terminal endonuclease domain protein of PA subunit of the viral polymerase to 100 μl of assay buffer in each reaction (final concentration of the protein is about 0.1 μM). 

### Docking simulation analyses of EGCG, ECG, EGC or EC with endonuclease domain of influenza A virus RNA dependent RNA polymerase *in silico*

     All molecular modeling studies were performed using Molecular Operating Environment (MOE; Chemical Computing Group, Quebec, Canada) software [12]. The X-ray crystallographic structure of the endonuclease domain of PA subunit of influenza A virus RNA dependent RNA polymerase (PDB ID: 3EBJ) was obtained from a protein data bank [6] [8]. This enzyme was prepared for docking studies in which (i) the ligand molecule was removed from the enzyme active site; (ii) hydrogen atoms were added to the structure with a standard geometry; (iii) the structure was minimized using a MMFF94s force-field; (iv) MOE Alpha Site Finder was used for active site searches within the enzyme structure and dummy atoms were created from the obtained alpha spheres; and (v) the obtained model was then used in AS_Dock program (Ryoka Systems Inc.).

## Results

     In our current study, we focused on the endonuclease activity of influenza A virus RNA dependent RNA polymerase and investigated whether green tea catechins inhibit this activity directly using the purified system. We first performed endonuclease assays by incubating the recombinant N-terminal domain (1- 220 residues) of the PA subunit with 0.1, 1, 10, and 100 μM of EGCG (Fig. 2a). We found from this analysis that EGCG inhibits the endonuclease activity of the PA N-terminal domain at a dose of 10 μM (Fig. 2a). This is the first demonstration of the inhibition of influenza A virus endonuclease by a green tea catechin.     To analyze the structure-function relationship of EGCG with respect to the inhibition of the endonuclease activity of influenza A RNA polymerase, we next determined which of its chemical groups is required for this effect. We used several catechins and related chemicals in this analysis i.e. EGCG, ECG, EGC, EC and gallic acid (GA). In addition to EGCG, ECG also showed inhibitory activity towards the endonuclease (Fig. 2b). On the other hand, catechins such as EGC, EC (without galloyl group) and GA (galloyl group only) showed weaker or no inhibition activity compared with EGCG and ECG (Fig. 2b). These structure-function relationships of the catechins indicated that the galloyl group is essential for full inhibition activity (Fig. 1). EGC and GA showed weak inhibition activity and EC showed no activity (Fig. 2b), suggesting that a hydroxy group on the catechins determines the strength of the endonuclease inhibition. Although the exact binding site and the mode of interaction between catechins and the endonuclease domain have not yet been resolved, we predicted that the galloyl group of EGCG attaches to an active pocket of the endonuclease domain of influenza A virus RNA dependent RNA polymerase.

     Our initial findings prompted us to investigate how green tea catechins bind to and fit with the active pocket of endonuclease domain of influenza A RNA polymerase. We thus performed in silico docking simulation analyses of this interaction at the tertiary structure level using Molecular Operating Environment (MOE) software [12]. The results showed that EGCG almost fits with and fills in the active pocket of the endonuclease domain of influenza A virus with its galloyl group (Figs. 1 & 3ab, left panels), whereas the binding of EGC produces a larger gap in the pocket (Figs. 1 & 3ab, the right panels). The difference between these two catechins is the presence of a galloyl group, suggesting the importance of this moiety for binding to the endonuclease active pocket. Calculations of the binding energy using docking simulations revealed lower values for EGCG and ECG compared with EGC and EC (Table 1), further indicating the importance of the galloyl group for more stable binding to the endonuclease. In addition, the hydroxyl groups of the galloyl group appear to attach with hydrophilic surface of the pocket of the protein (Fig. 3b). EGCG also has eight hydroxyl groups (Fig. 1) which seem to contribute to this binding via hydrogen bonding to the endonuclease domain protein (Fig. 3b).     These results are of great potential utility both for the design of novel anti influenza drugs, and chemical modifications of catechins to produce more potent effects against this virus. 

## Discussion

     East Asian people have been drinking green tea for about one thousand years, which underpins its safety for human consumption. Hence, it is reasonable to assume that the risk of any adverse effects of catechins and catechin-based drugs should be reduced compared with other possible compounds. Since the enantioselective synthesis of EGCG has been previously demonstrated [13], this compound can also be easily modified using organic chemistry methods, and catechin compounds can also now be synthesized chemically in large quantities. The results of our current in silico docking analyses provide information that will be of use in refining putative anti-influenza A drugs based on catechins by chemical modifications to fill in remained gaps of the pocket more tightly.     Song *et al.* showed that catechins inhibit the viral neuraminidase in the crude system and concluded that the effect is via the membrane [11]. This suggests that catechins are very unique chemicals to have dual function to inhibit both the viral neuraminidase and the endonuclease of the RNA polymerase. The RNA polymerases are more conserved among the viral strains compared to the neuraminidases. Thus, refinement of catechins based on our result will produce anti-influenza drug effective to wider strains of the virus.     Taken together, we propose from previous information and our current data that EGCG-based drug design has the great potential to produce a more effective and safer anti-influenza drug. 

## Acknowledgements

     The influenza (A/PR/8/34) RNA polymerase PA plasmid, pBMSA-PA, was provided by the DNA Bank, RIKEN BioResource Center (Tsukuba, Japan; originally deposited by Dr. Susumu Nakada) with the support of National Bio-Resources Project of the Ministry of Education, Culture, Sports, Science and Technology, Japan (MEXT).

## Funding information

     This work was supported in part by a Grant-in-Aid for Scientific Research from the Ministry of Education, Culture, Sports, Science, and Technology, Japan.

## Competing interests

     The authors have declared that no competing interests exist.

## Footnote

     Yuma Iwai, Hironobu Takahashi and Dai Hatakeyama are contributed to this work equally.Takashi Kuzuhara is a corresponding author. Tel.: +81 (88) 602-8477; Fax. +81 (88) 655-3051.  E-mail address: kuzuhara@ph.bunri-u.ac.jp.

## Figures

**Figure:**
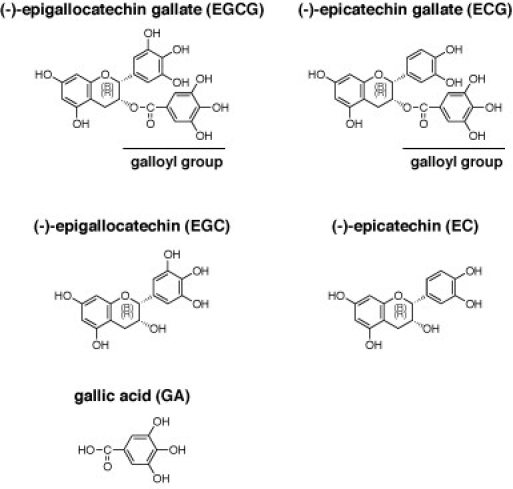

**Fig. 1** Chemical structures of the green tea catechins. The galloyl group is indicated.**Fig. 1** Chemical structures of the green tea catechins. The galloyl group is indicated.

**Figure:**
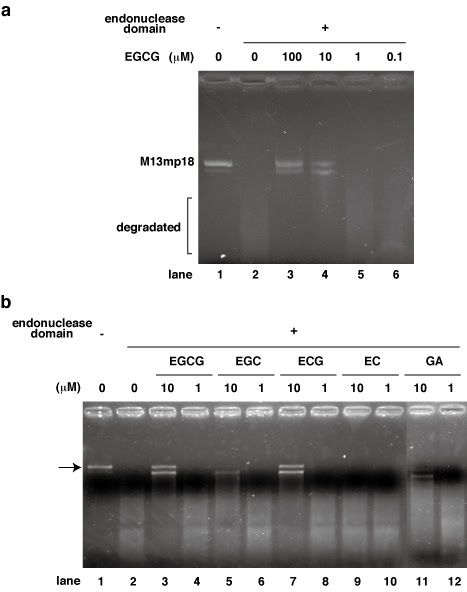

**Fig. 2**

**a) **Effects of EGCG upon the endonuclease activity of the N-terminal domain of the PA subunit of influenza A virus RNA dependent RNA polymerase.  The recombinant N-terminal domain of PA was added to each reaction at 0.35 μg/ 100 μl and a zero control (no PA domain added) was also assayed.  EGCG was added (μM) as follows: 0 (lane 2), 0.1 (lane 6), 1 (lane 5), 10 (lane 4) or 100 (lane 3).  M13mp18 was used as the substrate.

**b) **Effects of various green tea catechins upon the endonuclease activity of the N-terminal domain of the PA subunit of influenza A virus RNA dependent RNA polymerase.  The recombinant N-terminal domain of PA was added to each reaction at 0.35 μg/ 100 μl and a zero control (no PA domain added) was also assayed.  EGCG, EGC, ECG, EC and GA were added (1 or 10 μM).  M13mp18 was used as the substrate and the results were reproducible.  

**Figure:**
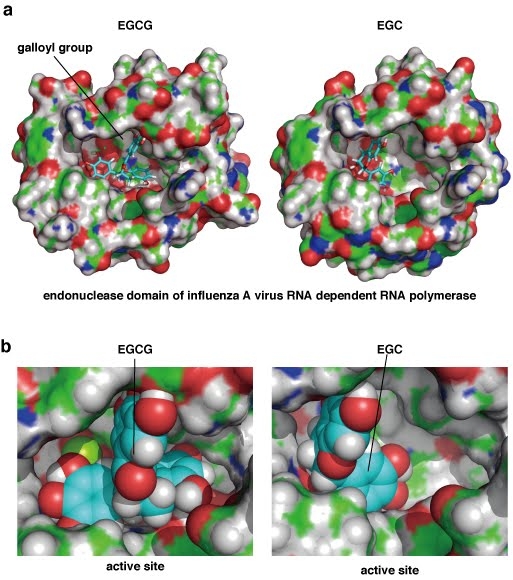

**Fig. 3 a**) Simulation analysis of the docking of green tea catechins with the endonuclease domain of influenza A virus RNA dependent RNA polymerase. Left panel: docking between EGCG and the endonuclease. Right panel: docking between EGC and the endonuclease. Green, red, blue and white colors indicate carbon, oxygen nitrogen and hydrogen atoms in the endonuclease domain of influenza A virus RNA dependent RNA polymerase, respectively. The light blue color indicates carbon atoms in the EGCG or EGC compound. **b**) The active pocket of the influenza A RNA polymerase endonuclease. EGCG and EGC are displayed in a sphere mode. Left panel: docking between EGCG and the endonuclease. Right panel: docking between EGC and the endonuclease. The green, red, blue, white and yellow colors indicate carbon, oxygen, nitrogen, hydrogen and sulfur atoms in the endonuclease domain of influenza A virus RNA dependent RNA polymerase, respectively. The light blue color indicates carbon atoms in the EGCG or EGC structures. These data have utility for future strategies to refine catechin-based drug design using an in silico binding simulation between PA and EGCG via the tertiary structure of PA.

**Table 1**  Summary of docking energy measurements. Lower numbers indicate more stable interactions.

**Table:** 

	EGCG	EGC	ECG	EC
Docking energy (kCal/mol)	-134	-112	-132	-109
galloyl group	+	-	+	-
